# Supporting Physical Activity for Informal Caregivers during and beyond COVID-19: Exploring the Feasibility, Usability and Acceptability of a Digital Health Smartphone Application, ‘CareFit’

**DOI:** 10.3390/ijerph191912506

**Published:** 2022-09-30

**Authors:** Kieren J. Egan, William Hodgson, Gennaro Imperatore, Mark D. Dunlop, Roma Maguire, Alison Kirk

**Affiliations:** 1 Department of Computer and Information Science, University of Strathclyde, Glasgow G1 1XQ, UK; 2 Physical Activity for Health Group, University of Strathclyde, Glasgow G1 1XQ, UK

**Keywords:** caregivers, innovation, research, co-design, interdisciplinary, digital health, participatory design, collaboration at distance

## Abstract

The COVID-19 pandemic has exposed how our global societies rely upon the care and support of informal (unpaid) caregivers: in the UK alone, there are an estimated 6.5 million informal carers. The caring role is not just precarious, it is often associated with high levels of stress, poor/deteriorating health and crisis points (hospitalisations, worsening of health). Fittingly, there has been much research in recent years focusing on mental health supports. A lesser explored area is physical health and physical activity. To address this, we conducted a real-world feasibility, usability and acceptability study of a novel codesigned digital health app for caregivers to improve levels of physical activity. Our study was designed to test the prototype app use for three weeks, following participants across questionnaires/in app data/qualitative data. Our findings (from 27 caregivers) highlights key knowledge gaps around physical activity—national guidelines were not reaching populations studies and behavioural change techniques hold promise to help support caregivers in the longer term. Our collective results support the acceptability, usability and feasibility of the Carefit app and warrant further investigation.

## 1. Introduction

The world population is ageing. By 2050 we can expect 2.1 billion individuals aged 60 years and over: a projected rise of 1 billion people (WHO, 2022). While increases in longevity need to be celebrated, they also pose societal issues for both achieving quality of life and delivering long term care. Around the world, much of our health and social care is delivered not by trained specialists, but by family and friends- informal carers [1,2]. Delivering care can be beneficial for informal carers (e.g., supporting community living, personal growth, resilience and altruism) but for many caregivers it contradicts societal ideals of healthy ageing [3,4]. For example, caregiving is associated with a wide array of negative short and long term consequences- many of which exacerbated by the COVID-19 pandemic [5]. Carers can be left feeling isolated, overworked and burnt out after delivering care for many years and/or many hours a week (including around the clock care). As the immediate impacts of the pandemic subside, critical questions are coming to the floor about how we build resilience in health and social care—including supporting caregivers [6,7].

While caregiving has been a topic of research interest for decades [8], the pandemic offers all in society a glimpse of the true value of care. The precise impact of the COVID-19 pandemic on caregivers may never be truly known. Prior to the pandemic, national U.K survey data suggested that 72% of carers experience mental ill health and 61% experience physical ill health due to caring [9]. Available data at a European level (data from 26 countries with 51,983 respondents) suggest that both anxiety and depression increased [10] whereas data from the UK suggest that many more people became involved in a caregiving role (almost half the population according to the UK Office for National Statistics [11]). Critically, for those who are already caregiving, pressures increased further [12,13]. Taken collectively, there is a question of whether the pandemic may initiate change so that we avoid the “revolving door” of crisis points to realise preventative and timely support and care [7].

To address these unmet needs, many digital health approaches that have emerged across support, care co-ordination, telehealth/diagnostics and digital care delivery [14]. In particular, a major focus has been made to target mental health (e.g., burden/anxiety/depression) through face to face, telephone and digital interventions [4,15,16]. Less established, has been the space of physical health for carers which has a long standing evidence base for benefits and is a clear area of public health concern [17,18]. Systematic review work in this area identified 14 studies, [19] (mainly face to face and telephone-based approaches) where intervention improvements for physical activity were seen across physical activity levels, distress, well-being, quality of life and sleep quality. Given that digital health approaches for physical activity in caregivers remains relatively unexplored, our aim was to evaluate the feasibility, acceptability and usability of a novel, user codesigned prototype ‘CareFit’ [20].

## 2. Materials and Methods

Prototype based evaluation is a widely used exploratory approach to gain early in-sight on usability [21]. We based our evaluation around a high-fidelity mobile prototype application in order to gain insights into both usability and potential usage patterns of informal carers. The use of a mobile high-fidelity prototype also supported remote distribution and user testing during COVID-19 personal contact restrictions.

*Inclusion/Exclusion details and ethical approvals:* Our inclusion/exclusion criteria were as follows. Participants must: (i) self-identify as an informal caregiver (caring for a family member or friend), (ii) be aged 18 or above, (iii) have normal or corrected to normal eyesight, (iv) be contemplating (starting to think about doing more physical activity) or preparing (being physically active occasionally and would like to become more active) to undertake physical activity and have the ability to undertake simple exercises such as arm raises or stretching. In addition, participants must have access to an Android smartphone, be comfortable installing apps, have access to the internet and be based in Scotland. Participants who had been advised by a clinician not to undertake physical activity or make any change in their present level of exercise were excluded from the study. Ethical permissions were obtained from the Strathclyde University Ethics Committee (UEC).

*Recruitment approach:* Participants were recruited using social media (e.g., Twitter, Facebook) from Carers Scotland and the University of Strathclyde. Our overall approach for participants (e.g., sample size) was informed by the WHO Monitoring and evaluation digital health interventions framework [22]. The 3-week study commenced on 12 October 2020, during the COVID-19 pandemic where there was no formal “lockdown” instructed (i.e., order to stay at home to save lives) from either the Scottish or UK governments.

**Figure 1:**
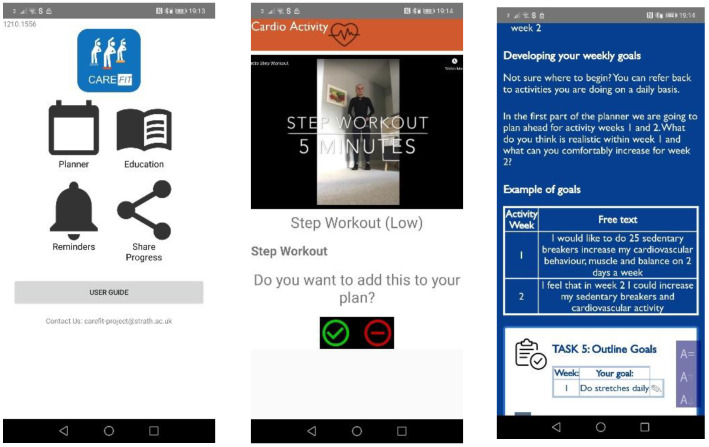
Visuals of the prototype used within the “CareFit” 3-week test study including the Main Menu (**Left**), Physical activity (**Middle**) and educational section (**right**).

*Description of intervention:* the components of our intervention have been described previously [20] and included educational, physical activity and communication components, that were co-designed with a range of stakeholders including caregivers. The intervention was based on the Transtheoretical model (TTM) of behaviour change and specifically for caregivers in a contemplation or preparation stage of change, i.e., people who are not active and meeting physical activity guidelines but showing motivation to be more physically active. Cognitive and behavioural strategies used within the Carefit app were tailored to these stage of change. See Figure 1.

Briefly this was comprised of:

- *Educational components:* including the following “stages”: (1) Welcome and Introduction (2) Physical activity: Beginners Guide” (3) “Relationships and” Physical Activity”, (4) “Managing time”, (5) “Goals and Rewards”, (6) “Physical activity and consequences” (7) “The Mind and body” and; (8) “Knowledge Quiz”.
- *Physical activity components:* (a weekly planner that allows planning for two weeks ahead and viewing of the week previous). The planner and physical activity options supports three different components of physical activity (cardiovascular activity, strength and balance, sedentary behaviour with a bespoke icon and individual screen for each). Videos for each component for caregivers were developed by colleagues at the University of Strathclyde who have previously worked with caregivers.
- *Communication elements* and an *online user guide.*

*Data collection and analysis:* Data were collected across three key formats- (i) online questionnaires, (ii) post use interviews and (iii) “in app” usage statistics.

(i) *Online Questionnaires:* We collected baseline information through questionnaires (using Qualtrics software, Seattle, WA, USA) that were provided to users in app. Questions covered basic demographic information (e.g., age, gender, number of years caring, number of hours caring per day, the council district of residence), details around health and wellbeing (e.g., medical conditions of the carer, current levels of physical activity), education level, work status alongside, motivators and barriers to physical activity (including decisional balance, self-efficacy, stage of change in the TTM). Our definitions of physical activity were based on UK government guidance where; (i) “meeting guidelines” equated to “at least 150 min/week of moderate physical activity, 75 min/week vigorous physical activity, or an equivalent combination of these”, (ii) “some activity” equated to “60–149 min/week of moderate physical activity, 30–74 min/week vigorous physical activity, or an equivalent combination of these”, (iii) “low activity” equated to “30–59 min/week of moderate physical activity, 15–29 min/week vigorous physical activity or an equivalent combination of these” and (iv) “very low activity” equated to “less than 30 min/week of moderate physical activity, less than 15 min/week vigorous physical activity, or an equivalent combination of these”. Optional interim feedback was possible also through a short free text response delivered mid-way through the 3-week test study. The follow up survey involved a ‘push’ of surveys in a similar manner. Users could provide feedback on free-text opinions of the app. We used the second Unified Theory of Acceptance and Use of Technology (UTAUT2) [23] to add clarity to the key strengths/weaknesses of the app and to help with future directions. The UTAUT2 is a well-established scale that hypothesises that expectancy, effort expectancy, social influence, and facilitating conditions are the determinants of behavioural intention or use behaviour [24].
(ii) *Telephone interviews:* We aimed to interview 6 to 10 participants, selected at random (using random function within excel). We used a semi-structured interview to explore themes as described in the study protocol. Interviews took no longer than 20 min and were recorded using an encrypted Dictaphone before being fully transcribed.
(iii) *In app data:* As each participant was assigned a unique identifier- we explored access and use of the educational and physical activity content within the app. This included data relating to the initiation and completion of the educational ‘stage’ (stored with a time stamp for each user). Physical activity elements included the type of activity planned and completed (across the three core elements of ‘cardiovascular’, ‘muscle and balance’ and ‘sedentary breaker’ activities). For physical activities we additionally stored information on which specific activity was undertaken (e.g., ‘muscle and balance exercise 1’, ‘cardiovascular exercise 2’, etc.). We did not record information on the weekly plans made by each user or record social/media communication elements from users within the app stored data.

*Development*: CareFit was developed by two members of our research group at the University of Strathclyde using an Agile methodology which consisted of incremental (mostly) bi-weekly sprints. After each sprint the app was evaluated by our team and our participants, all stakeholders contributed to determining which functionalities to add, remove or edit. The app was mostly developed using the Android SDK in Java; the education section, however, was developed in HTML/JS to ensure that educational text was presented in the best possible manner. CareFit was compatible with Android version 5.1 (API level 22) up to Android version 9.0 (Google, Google HQ, API level 28). User data was sent to a server hosted database for later analysis. The app was designed to ensure that data were collected at all times even if a user had no internet access while the app was being used. Most of the functionalities of the app were available offline with the exception of exercise videos. This was because it was decided to leverage YouTube both for hosting convenience purposes and to delegate video playing functionalities and subtitling to speed up development times.

*Data analysis:* Our interpretation of the acceptability, usability and feasibility of the ‘CareFit’ concept was based on an integrative mixed methods approach where we identified common themes across different data sources. For qualitative data, we used Braun and Clarke thematic analysis [25] where transcripts were systematically analysed by two different reviewers. Transcripts were coded and themes identified through the use of a coding tree. Quantitative data (e.g., in app data, likert scales) were explored using standard statistics, including frequency counts, percentages and standard deviations. The vast majority of feedback obtained is through likert scales hence no standard deviations are given. The exceptions to this were both the sedentary based question (where participants were asked to rate the portion of the day spent sedentary [where 10 equals 100% of the day]) alongside findings from the UTAUT2 scale.

## 3. Results

Sixty-eight carers expressed an initial interest in the study and where reasons for non-participation were declared, 2 carers were already regularly exercising and 5 carers did not have access to an android phone. Only 34 of these 68 caregivers returned consent forms. Where reasons for not joining study were given, 2 caregivers had to withdraw interest due to caring duties, and 4 had to withdraw interest due to a lack of android device. In total 28 carers consented to the study where one participant joined the study too late to receive the baseline measure and was therefore not included in analyses. In addition, 5 carers dropped out during the study leaving 22 carers registered on the app system. Follow up analyses integrate information across 6 follow up interviews, 22 carers ‘in app’ data, 16 mid-week surveys and 13 carers final study surveys alongside email feedback for troubleshooting/support.

Our initial demographics analyses (see Table 1) indicated that at baseline, our participants were predominantly female (85.2%), white (100%), urban based (70.4%) and geographically diverse (across 16 different Scottish council areas). Our participants varied in terms of educational backgrounds, although commonly educated to degree or equivalent (55.6%). Caregiving experience differed, but was more often considerable: 51.9% of our sample had been caring for 10 or more years, and 63.0% of carers spending 8 or more hours on their caregiving role.

Physical activity knowledge and experience at baseline was limited (see Table 2). A total of 15 respondents (57.7%) had not heard of the U.K. physical activity guidelines and 8 participants (30.8%) were “very aware” of the benefits of physical activity. Additionally, 15.4% of carers said that the COVID-19 pandemic had had “no impact” on their time for physical health and overall, and 48.2% of participants were in the “very low” category of activity (i.e., less than 30 min/week of moderate physical activity, less than 15 min/week vigorous physical activity, or an equivalent combination of these) category of activity levels). Respondents survey spent an average 54.3% of the day sedentary. The most commonly reported barriers to physical activities were (lack of) motivation, (73.1% of users), caring duties (61.5%) and lack of time (46.2%). We explored the specific motivations mentioned by caregivers: improving physical health, keeping fit and losing weight were prominent reasons given. At baseline, carers lacked both support and confidence for embarking on safe and effective physical activity, 61.5% of users felt that they were “not supported” whereas 11.5% caregivers felt “highly confident” towards physical activity. We did not statistically compare with our baseline and follow up measures as this was beyond the scope of the study aims (Table 3). However, data supported future investigation of outcomes around awareness of the physical activity guidelines, physical activity levels, and support/confidence levels.

### 3.1. Feasibility of the ‘CareFit’ App

We explored whether it was feasible to deliver the “CareFit” app, both from the viewpoint of the participants and as an overall prototype system. Thematic analysis of carer feedback from the surveys and interviews identified nine key themes. These were: it must; (i) be inclusive, (ii) be phone accessible, (iii) be time flexible, (iv) allow goal setting/planning ahead, (v) allow monitoring of physical activity progress, (vi) deliver interactive education on physical activity, (viii) motivate caregivers (e.g., support behavioural change) and (ix) facilitate connections to other people (See Table A1).

Each of these user needs was successfully demonstrated within our feasibility testing over three weeks. Our prototype demonstrated functionality, in part by the successful collation of the “in app” data across both educational and physical activity start and completion statistics where 22/27 caregivers (81%) completed a physical activity component and 19/27 (70%) completed an educational event. Use of the app centered around low intensity physical activities and many caregivers added routine caregiver activities as a physical activity undertaken. Overall, 50% of activities performed were in the “Cardio” category with the remainder split evenly between “Muscle and Balance” and self-entered “Other” category (these included, for example, caring duties, household tasks, and dog walking). Of the 19 users who completed an educational task, 11 completed at least half the sections while 4 users completed all sections. Users tended to work through sections in order with some skipping individual section, as such the first two sections had the highest completion rates. Within the sections we had included small reflective tasks, 13/27 (48%) of the users completed at least one of these optional functional tasks (e.g., writing a pros and cons list). We analysed task completion rates against education section completion rates for individuals but found only very weak correlation (R^2^ = 0.33).

The overall concept of the app was praised by some participants as addressing the barriers around time pressures:

*“I can now fit my exercise around my caring duties with my son” Carefit Participant.*

There were also notable challenges to deploy the ‘CareFit’ app in real world settings. In particular, the “real time” nature of this study meant application updates needed to be “pushed” to the Google Play Store and users had to update their app manually (where this does not happen automatically). There were also a small number of occasions where some users could not get specific elements of the app working (see below). Nevertheless, the vast majority of our participants were able to successfully download the app, complete at least one educational activity or physical activity (see acceptability and usability).

### 3.2. Overall Acceptability and Usability of the ‘CareFit’ App

We explored the data around both the acceptability and usability of the app using the three data streams as outlined in the methods, and by examining out dropout statistics. We identified that from the 27 active users, 22 participants used both the education and physical activity sections of the app within the three weeks (equating to a 22% drop-out rate). Of these 22 participants, 16 (72%) successfully completed a form of further feedback (e.g., surveys, interviews, in app data collected) that we could use to explore acceptability and usability. Overall, many respondents were positive about overall use of the app (See Table A1), and the majority of feedback received suggested that fundamental concepts that arose in our early generative codesign stages were paralleled in real world settings.

*“Overall I enjoyed using the app and found it easy to use” Carefit Participant.*

Such findings were also indicated through the use of the UTAUT2 Likert scale feedback (Table A2) where the domains of effort expectancy and facilitating conditions were areas of particular approval from respondents (e.g., *I find CareFit easy to use*). The components of “Hedonic Motivation” (e.g., *Using Carefit is fun/enjoyable*) were also largely supportive of app use, and carers demonstrated through “Facilitating Conditions” that they had both the resources and knowledge to use CareFit successfully.

### 3.3. Barriers and Facilitators for Use of ‘Careft’

Our qualitative analysis identified nine key themes for barriers and enablers for the use of the CareFit app: (i) technology/phone delivery approach, (ii) instructions and guidance, (iii) technology experience of the carer, (iv) personalization and flexibility, (v) planning ahead, measuring progress and changing needs, (vi) wider behavioural change, (vii) individual carer circumstance alongside (viii) self-awareness of physical and mental health and (ix) social connections. See Table A3.

The majority of qualitative feedback regarding the app identified our approach aligned as supportive and empowering: a number of participants mentioned that they would like to use it for longer and during the winter months. Participants could identify ways to gauge their own progress/improvement—e.g., losing weight, or, building up to a new physical activity level relative to where they were starting from. Many carers were clear that physical and mental health awareness and motivations were elements that were successfully delivered within the context of the app. Advantageous was; the informal style of the app and the encouraging delivery of physical activity elements—including the specific instructor delivering physical activities (e.g., in a local indoor setting within the home and a local accent).

*“The app was gently telling me to look after myself. I loved the instructor she just cared.” Carefit Participant.*

Caregivers differed with respect to communication and supports. Our user group were starting with different technology experiences/expectations, physical activity knowledge/levels and social networks. For example, a number of caregivers commented that physical activities are something they would like to keep private whereas others saw real value in sharing their experience with immediate friends and family. However, it was also apparent that many participants could not see the role/value for sharing their activities with social media contacts.

*“The family were very helpful and enjoyed doing some of the exercises together. This is something we haven’t done a lot together recently.” Carefit Participant.*

Caregivers accordingly progressed through materials at different rates. Other examples of attributes of the app that were useful to some users but not others included the reminders functionality where responses ranged from being heavily reliant, to no/little use. There were also conflicts between user needs—some users were looking for more complex physical activities, however the majority of respondents identified value in the style and detail of the materials presented. While many users enjoyed the freedom that having the materials on a mobile phone provided, some users suggested that greater interoperability and automated data collection would suit their own circumstance better.

### 3.4. Future Perspective

Finally, we explored three key themes around the future use of the “CareFit” app with participants, particularly around extensions of the project (human and technology based) and what consumer price point for use would be considered for a full version to be implemented. A number of participants commented that making sure achievements are always recognised was a clear area for further development- and the use of automated/wearable technologies approaches could be suitable (e.g., GPS and pedometer/accelerometer). Carers identified that the resource would connect well to points of contact within the health and social care infrastructure such as General Practitioners, visiting healthcare professionals, or care support groups. The concept of future payment appeared largely acceptable (both personally and health and social care supported)- and a suitable price point may work as a one-off purchase fee or a subscription model (e.g., £1 a week).

## 4. Discussion

**Figure 2:**
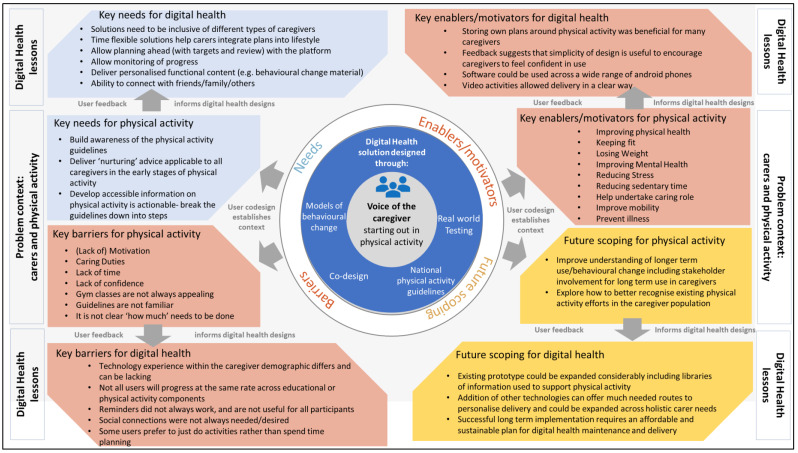
Key themes identified from the development of ‘CareFit’, including the core needs of users, barriers, enablers and motivators and future scoping.

The COVID-19 pandemic has been an unprecedented challenge to our global health and social care systems [26]. It is clear our societies around the world depend on those who care, and those that care are at risk of poor health [13,27,28,29,30]. As normality returns, key questions must be asked to mitigate the impacts of ongoing underlying ‘syndemics’ [31]. Here, we have evaluated a novel digital health and wellness innovation through an authentic co-design process to appraise the feasibility, acceptability and usability. We identified that many experienced caregivers (e.g., caring for 10 or more years) have little/no awareness of physical activity guidelines. Many in this group—when given the right support and tools– found making positive changes towards their health and wellbeing achievable. Therefore, this collective work offers an advancement of current understanding of barriers, enablers and a future perspectives for technology to support physical activity in caregivers (see Figure 2).

Informal caregivers are a complex group to understand and support in a timely manner [32,33]. While there is a continued challenge to support all vulnerable population groups to stay physically active [34], our work here suggests that relatively simple public health messages on physical activity are not reaching caregivers—who are already highlighted as a population at risk of poor physical and mental health. As a society it is critical that we do our utmost to meet the information needs of caregivers- and also to provide accessible routes to both physical and mental health actions—prior to crisis points (e.g., hospitalisaitons/rapid declines in health). For these reasons, the concept of ‘Carefit’ fits a narrative of early preventative measures to support caregiver health and prioritizes those who may have been caregiving for many years and are looking to change habits. Taken collectively, the lens of physical activity offers alignment with concepts around salutogenesis: ‘creating’ health and wellness opposed to focusing on risks [35].

The feasibility of our system and approach was demonstrated by both feedback and data from participants. Our Android application functioned well across three weeks of “real world” use across Scotland where the vast majority of our participants accessed physical activity videos or educational sections. We were not prescriptive about how much the intervention should be used (other than suggesting education materials could be used in the first week) instead, presenting the physical activity guidelines and encouraging users to make an achievable plan to move towards these. Nearly all our participants started the study stating that had self-motivational reasons to join the study including to improve physical health, lose weight or improve mobility. Throughout, motivators and barriers to physical activity aligned with other literature of non-carer groups elsewhere [36,37] with the notable exception that “Caring duties” were often a leading barrier to starting out in physical activity for our participants. Our carer participants were clear about their expectations of what excellence looks like for digital solutions to overcome such barriers—solutions must be accessible, accommodating, inclusive and empowering- recognizing the caregiving role. In terms of acceptability and usability, participants enjoyed simplicity in design and the accessible manner of the written educational materials and physical activity videos based around TTM and physical activity guidelines. There are improvements that can be made around the Graphical User Interface (GUI) in any future versions. Carers valued recognition of their caregiving tasks through the application and a delivery that was culturally appropriate (e.g., local accent).

Taken the above collectively, this work presents an argument that “off the shelf” solutions (e.g., GPS, step-based apps) or high intensity fitness apps alone cannot fully recognize the underlying motivations and/or achievements of carers- particularly those starting out in physical activity. More personalized, motivational approaches offer more targeted but ‘actionable’ and evidence-based guidance for those who are contemplating/or starting out in the process of behavioural change. While digital health approaches offer many strengths, our experience highlights that physical activity interventions have considerable responsibilities about ensuring careful interpretations of guidelines. For example, there remain ambiguities around how much sedentary time or muscle and balance activity is needed [38] and systematic review work in the area demonstrates how varied interpretations of regular physical activity can be found [19]. There is also a growing body of research emerging from the COVID-19 pandemic and physical activity interventions that holds potential for research and implementation. For example, Gonçalves (2022) [39] conducted a scoping review and identified a growing interest in the transtheoretical model for recent intervention study designs and that general physical activity counselling interventions generally increased physical activities of participants.

Despite many successes of CareFit, there are a number of limitations within this work which should be taken into consideration. First and foremost is that we are cognisant of the considerable challenge that both long term behavioural change [40] and implementation [41] pose, however this work still represents a strong indication of future potential. There are also multiple approaches possible to appraise acceptability, usability, and feasibility- but wherever possible we opted to combine our data sources (qualitative interviews, app use and questionnaires) to address such components comprehensively including using standardized measures and instruments (e.g., UTAUT2). We have also discussed here the diversity of caregivers-it is not conceivable that any single solution is capable of targeting and sustaining behavioural change for all- we specifically targeted carers who were starting out in physical activity (i.e., likely to be the least well served by existing technologies) but there remains much scope to extend the reach of digital health supports for caregivers across the physical activity spectrum. It is without question that our findings highlight that personalization is possible but remains challenging to deliver in practice. Previous work has also found that what is encouraging for one carer can be off-putting for another [14]. Some carers did not engage with the platform, but this is somewhat expected, and our prototype has been tested during a trying time (i.e., a global pandemic). Equally, long term sustainable change is of more value to all stakeholders opposed to short term benefits. Crucially, this work was designed as a rapid response 3-week study. Further work is required to expand the concept, functionalities and support longer term behaviour change and effects/impacts. As with any ‘real world’ prototype there were also a number of technical challenges dealt with as they arose- one of these was reminding users to update their phones which we suspect limited feedback. Nevertheless, the integrative mixed methods approach identified that the majority of our participant responses found value in the concept of a digital health approach to support regular physical activity in carers from home during and beyond the COVID-19 pandemic.

## 5. Conclusions

Across the world, millions of caregivers continue to support others with day-to-day care, often at the expense of their immediate physical and mental wellbeing alongside long term health. Here, we set out to understand the acceptability, usability and feasibility of an innovative concept to support physical activity in caregivers in a rapid response project in relation to the COVID-19 pandemic. Designed as an inclusive, motivational and evidence-based tool (including extensive input with carers), ‘CareFit’ not only demonstrated feasibility but was well accepted and easily used by participants. There remains clear (and basic) knowledge gaps between physical activity guidelines and caregiver populations. Such findings not only add to the sparse literature of physical activity solutions for caregivers- they seed a narrative around what holistic health and wellness could look like for carers as we learn to live with COVID-19 and navigate future pandemics.

## Figures and Tables

**Table 1:** Demographic information of participants collected at baseline.

**Gender (*n* = 27)**	***n***	**%**	**Location (*n* = 27)**	***n***	**%**
Male	4	14.8	Edinburgh city	3	11.1
Female	23	85.2	North Lanarkshire	3	11.1
**Ethnicity (*n* = 27)**			Inverclyde	3	11.1
White	27	100	Glasgow city	2	7.4
Mixed/multiple ethnic groups	0	0	Perth and Kinross	2	7.4
Asian/Asian British	0	0	Angus	2	7.4
Black/African/Caribbean/Black British	0	0	South Ayrshire	2	7.4
Other ethnic group	0	0	East Lothian	2	7.4
**Education level (*n* = 27)**			Fife	1	3.7
Degree or equivalent	15	55.6	South Lanarkshire	1	3.7
Higher education	4	14.8	Highland	1	3.7
SVQ	3	11.1	Falkirk	1	3.7
School qualifications	3	11.1	Dundee City	1	3.7
Other qualifications	2	7.4	North Ayrshire	1	3.7
**Age group (*n* = 27)**			Moray	1	3.7
18 to 24	1	3.7	East Renfrewshire	1	3.7
25 to 34	2	7.4	**Setting (*n* = 27)**		
35 to 44	4	14.8	Urban	19	70.4
45 to 54	12	44.4	Rural	6	22.2
55 to 64	7	25.9	Not sure	2	7.4
65 to 74	1	3.7			
**Years caregiving (*n* = 27)**					
1 year or less	2	7.4			
Up to 2 years	0	0			
Up to 3 years	3	11.1			
Up to 10 years	8	29.6			
10 year or more	14	51.9			
**Hours caregiving per day (*n* = 27)**					
Up to 4 h	3	11.1			
Up to 6 h	4	14.8			
Up to 8 h	3	11.1			
8 h and more	17	63.0			
**Work status (*n* = 27)**				***n***	**%**
Caregiving has not affected my working/studying hours				7	25.9
Yes, caregiving has caused me to reduce my working/studying hours				5	18.5
Caregiving has caused me to give up work/study				8	29.6
I do not currently work/study and did not have to give up work/study due to my caregiving role				7	25.9

**Table 2:** Physical activity levels and views of participants at baseline.

**Familiarity with physical activity guidelines (*n* = 26)**	***n***	**%**	**Awareness of the benefits of physical activity (*n* = 26)**	***n***	**%**
Not heard of these	15	57.7	I am not aware of these	1	3.9
Heard of it, but very unfamiliar	6	23.1	Aware of these, but very unfamiliar	5	19.2
Heard of it, but mainly unfamiliar	3	11.5	Aware of these, but mainly unfamiliar	7	26.9
Broadly familiar	1	3.9	Broadly Aware	5	19.2
Very Familiar	1	3.9	Very aware	8	30.8
**Motivation for undertaking physical activity (*n* = 26)**			**Barriers to activity (*n* = 26)**		
To improve my physical health	23	88.5	Motivation (lack of)	19	73.1
To keep fit	18	69.2	Caring duties	16	61.5
To lose weight	18	69.2	Lack of time	12	46.2
To improve my mental wellbeing	18	69.2	COVID-19 restrictions	9	34.6
Reduce stress	15	57.7	Finances	8	30.8
To reduce my sedentary time	15	57.7	Weather	6	23.1
To help undertake my caring role	14	53.8	Lack of equipment	6	23.1
Improve my mobility	14	53.8	Confidence	5	19.2
To prevent illness	12	46.2	Physical space/environment	5	19.2
Improve posture	8	30.8	Others	3	11.5
Improve balance	7	26.9			
Other	1	3.85			
**Support (*n* = 26)**			**Confidence (*n* = 26)**		
Highly supported	1	3.85	Not confident/No confidence	8	30.8
Moderately supported	5	19.2	Slight confidence	6	23.1
Slightly supported	4	15.4	Moderate confidence	9	34.6
Not supported	16	61.5	High confidence	3	11.5
**Physical activity levels (*n* = 27)**			**Sedentary behaviours (*n* = 26)**		
Meets Guidelines	3	11.1	Average proportion of day spent sedentary		5.43
Some activity	2	7.4	SD of this value		2.18
Low activity	9	33.3			
Very low activity	13	48.2			

**Table 3:** Physical activity levels and views of participants at follow up.

**Familiarity with activity guidelines (*n* = 13)**	***n***	**%**	**Awareness of the benefits of physical activity (*n* = 13)**	***n***	**%**
Not heard of these	3	23.1	I am not aware of these	0	0
Heard of it, but very unfamiliar	6	46.2	Aware of these, but very unfamiliar	1	7.69
Heard of it, but mainly unfamiliar	1	7.7	Aware of these, but mainly unfamiliar	1	7.69
Broadly familiar	3	23.1	Broadly Aware	7	53.9
Very Familiar	0	0	Very aware	4	30.8
**Motivation for undertaking physical activity (*n* = 13)**			**Barriers to activity (*n* = 13)**		
To improve my physical health	11	84.6	Motivation	9	69.2
To keep fit	9	69.2	Caring duties	9	69.2
To help me undertake my caring role	8	61.5	Lack of time	7	53.9
To prevent illness	8	61.5	Weather	5	38.5
To improve my mental wellbeing	7	53.9	COVID-19 restrictions	5	38.5
Reduce stress	7	53.9	Confidence	4	30.8
To lose weight	6	46.2	Others	3	23.1
Improve my mobility	6	46.2	Finances	2	15.4
To reduce my sedentary time	6	46.2	Physical space/environment	2	15.4
Improve balance	4	30.8	Lack of equipment	2	15.4
Improve posture	4	30.8			
Other	2	15.4			
**Support (*n* = 13)**			**Confidence (*n* = 13)**		
Highly supported	4	30.8	Not confident/No confidence	0	0
Moderately supported	1	7.7	Slight confidence	3	23.1
Slightly supported	3	23.1	Moderate confidence	3	23.1
Not supported	5	38.5	High confidence	7	53.9
**Current physical activity levels (*n* = 13)**			**Sedentary behaviours (*n* = 13)**		
Meets Guidelines	4	30.8	Average proportion of day spent sedentary	4.89	
Some activity	1	7.7	SD of this value	2.00	
Low activity	3	23.1			
Very low activity	5	38.5			

## Data Availability

Anonymous summary data will be available from the lead researcher’s PURE database (please email kieren.egan@strath.ac.uk for further information).

## Appendix A

**Table A1:** Qualitative feedback of the needs of participants around feasibility, accessibility and usability of ‘CareFit’.

Need/Requirement	Example Quote 1	Example Quote 2
*Feasibility*	*Assess whether the digital health system works as intended in a given context*	
Inclusive of many caregivers	The app has also encouraged me to get out for longer walks which has been difficult due to COVID and caring.	I can now fit my exercise around my caring duties with my son.
Phone accessible	It was great having these on my phone as I could take this with me anywhere in the house.	It is really tough for carers just now not being able to get out, but this app showed me the things I can do in my own house.
Time flexible	I would do the cardio in the morning and some strength ones in the afternoon which fitted into my routine.	So, the sedentary ones were great you could be sitting there with the person you are caring for and do the exercise without having to go away somewhere else to do the exercise.
Monitoring physical activity progress	The fact that the exercises were gentle, and you could build up made them more engaging.	They motivated me to be more active and defiantly kept me going
Goal setting and planning ahead.	I would have preferred to just click a button, do the exercise and it was recorded onto the planner rather than the other way around. I don’t know if that would work for everyone, but I am retired and have a set routine and would just like to record my activity as I did it rather than have to pre-plan it.	I would have liked the planner to be able to add more than 3 options if I decided to do more activities. I couldn’t record all the various activities I was doing if they were more than 3 a day.
Delivery of education materials on physical activity	This can be very useful at this time as most health care is out of reach for a lot of people just now because of COVID with most clinics cancelled. Carers like me with underlying health conditions feel isolated just now and your educational section was very useful.	I think the education part of it is very important for people that haven’t started that process and don’t understand that there is a definite link between the amount of exercise that you need to do and general fitness and overall health.
Functions to motivate behavioural change (e.g., planner, videos, text materials)	The app was gently telling me to look after myself. I loved the instructor she just cared.	The app has also encouraged me to get out for longer walks which has been difficult due to COVID and caring.
Facilitate connections to human supports (e.g., friends/family/other carers)	No not really I am quite a private person. I did though send an email to my son saying what I had done, and this brought us closer together.	I do not do social media so wasn’t sure who I should share my stuff with.
*Acceptability and usability*	*Would caregivers be willing to use it-and is the digital health system used as intended?*	
Overall	Overall I enjoyed using the app and found it easy to use.	This app has encouraged me to be more active.
Presentation of educational materials	It was well written though and I kept going back over it. I liked the graphics which helped break-up the writing.	I found the reading of the education section really good. All the background and reasons for doing things was very helpful.
Reminders	I was trying to set a reminder for the following day, but it would only come up the day before.	It only appeared to work on the day I set it but not in the future.
Planner	I used the planner to measure what I had done and found this good to track my activities.	This app has encouraged me to be more active.
Physical activities and videos	The videos were good but I think having the ability to make them bigger would have helped.	Then in the second week I went in I must admit that I don’t think I pressed the start button properly when I was doing exercise as often as I was doing or even at all.
Share functionalities		I didn’t really understand who I was to share the information with friends, relatives etc.

**Table A2:** Summary of participant responses from the Unified Theory of Acceptance and Use of Technology (UTAUT) 2 scale. Total number of respondents = 13.

Performance Expectancy	Mean	SD
I find CareFit useful in my daily life	4.92	1.80
Using CareFit increases my chances of achieving things that are important to me	4.85	1.72
Using CareFit helps me accomplish things more quickly	4.23	1.69
Using CareFit increases my productivity	4.23	1.54
**Effort expectancy**		
Learning how to use CareFit is easy for me	5.54	1.45
My interaction with CareFit is clear and understandable	5.25	1.29
I find CareFit easy to use	5.69	1.25
It is easy for me to become skilful at using CareFit	5.69	0.95
**Social Influence**		
People who are important to me think that I should use CareFit	3.38	1.85
People who influence my behaviour think that I should use CareFit	2.69	2.06
Social Influence—People whose opinions that I value prefer that I use CareFit	2.85	2.38
**Facilitating conditions**		
I have the resources necessary to use CareFit	5.69	1.44
I have the knowledge necessary to use CareFit	5.85	0.80
CareFit is compatible with other technologies I use	4.46	1.81
I can get help from others when I have difficulties using CareFit	5.23	1.64
**Hedonic Motivation**		
Using CareFit is fun	4.69	1.70
Using CareFit is enjoyable	4.85	1.86
Using CareFit is very entertaining	4.31	1.75
**Habit**		
The use of CareFit has become a habit for me	4.69	2.18
I am addicted to using CareFit	2.15	1.63
I must use CareFit	2.46	1.51
Using CareFit has become natural to me	3.69	1.65
**Behavioural Intention**		
I intend to continue using CareFit in the future	4.77	2.09
I will always try to use CareFit in my daily life	4.31	2.21
I plan to continue to use CareFit frequently	4.62	1.98

**Table A3:** Qualitative feedback of the barriers and enablers for use of “CareFit”.

Barriers and Enablers	Facilitator/Enabler Example	Barrier Example
**Current**		
Instructions and guidance	At the beginning I found the educational section very confusing and a lot of information. It was only when I finished it that I had a better understanding. It was great though being able to go back and look over the educational material again.	I had completed the educational section early and wasn’t sure if I should start the activities. You clarified that I could start using the planner at that stage which wasn’t clear in the instructions.
Personalization and flexibility	I also loved the flexibility and length of the exercise videos. Five mins was ideal and if you ran out of time you could just stop and still feel you had done something. If the videos had been any longer I would have struggled to do the exercises but the times on the app were perfect and really motivated me to be more active.	I would have preferred to just click a button, do the exercise and it was recorded onto the planner rather than the other way around. I don’t know if that would work for everyone, but I am retired and have a set routine and would just like to record my activity as I did it rather than have to pre-plan it.
Planning ahead, measuring progress and changing needs	To Learn and I have—my own routines at last. Been to Gym with variety of staff taking class not so good. This was consistent and I was able to Learn the routines and the options, but I MISS THE APP now the trial ended. It was helpful.	Easily forgot about it as reminders didn’t work and as I never saved it to my home screen it was out of sight. The videos were too much for me to compute, some went through several versions of exercise and I got lost in what the first one was
Technology experience of carer	I actually found it ok and did it myself which for me was a surprise as I am not good with these things.	I think it was just from my point of view that I felt had it been something I was more used to an integrated app. I use Fitbit quite a lot for example and you have got Fitbit on and you start going for a walk it recognises that you are going for a walk and starts measuring distance, stride, steps and so on.
Social connections	The family were very helpful and enjoyed doing some of the exercises together. This is something we haven’t done a lot together recently.	This is just for me and I don’t feel the need for support from others. Others might find this useful, but I don’t feel the need to share my personal activities with Joe Blogs in Largs.
Wider behavioural change	Fitness level and energy level plus confidence to look after myself for a change its a shift in outlook. The education part lifted me to feeling I needed this and deserved this. Love the Schedule and the programming.	When you are out exercising you are worried about meeting people.
Individual carer circumstance	It was great having these on my phone as I could take this with me anywhere in the house.	This can be very useful at this time as most health care is out of reach for a lot of people just now because of COVID with most clinics cancelled. Carers like me with underlying health conditions feel isolated just now and your educational section was very useful.
Self-awareness of physical and mental health	I have been trying to lose weight and this app has got me thinking about not only exercise but my diet.	You get wrapped up in other things and sometimes forget about yourself.
Technology/phone delivery approach	I loved having it on my phone so I could do the exercises when and where I wanted.	Reading so much information on the phone screen was also difficult.
**Future scoping/sustainability**		
Extend the recognition of further carer physical activities	I don’t know if there are any that don’t use the phone metrics to gauge what it is that you are actually doing. So whether if you are going for a walk or whether its anything that you are doing physical activity that you might be doing like lifting weights or doing housework or whatever it is that you are using to get your exercise or burn calories then.	“Felt extremely supported. The Healthcare professional visiting said a lot of carers she supports would like to know about CareFit’.
Integration with other technologies	Linking it into the phone metrics would help. GPS or steps. Most smartphones have these facilities and would be a great addition to the app.	I tried to link my phone with my TV, but it didn’t work. Having that facility to link with my TV would have been great. I found this difficult when doing some of the exercises such as against the wall or on the floor. I had to carry my phone and found it difficult to see the screen.
Pricing	If it was a modest fee then yes. I also use myfitnesspal and happy to pay for the additional services. If it was about £1 a week then I think that is reasonable.	It would depend if it was a one off or a monthly payment. I did pay recently for a type of house organising app. I think it was about £4.99 as a one-off payment. I found this useful so didn’t mind paying for it. I would be happy to get access of this app through either a GP or care support group. The human interaction is really important for carers as it can be very lonely on occasions.
